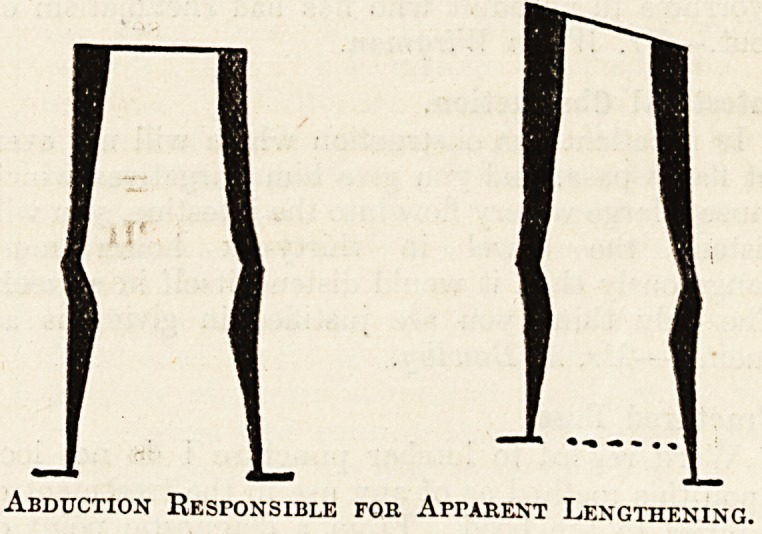# Inequality in the Length of the Lower Extremities

**Published:** 1910-08-13

**Authors:** 


### SURGERY.

### INEQUALITY IN THE LENGTH OF THE LOWER EXTREMITIES.

Assuming one limb to be normal, inequality in the length of the two limbs may be the result of lengthening or shortening of its fellow. The extent of this difference can only be estimated by measurement, and the points usually selected for this purpose, so far as the lower extremities are concerned, are the anterior superior spine and the tip of the internal malleolus. Owing to the fact that the spine is not

Shortening Masked by Posture.

Lengthening Masked by Posture.

situated on the limb itself and to the configuration of the neck of the femur it follows that the length of the limb will vary slightly according to the posture assumed. Thus the measurement between these two points will be less in the abducted than in the straight position. The difference, however, is so slight that for practical purposes it may be ignored. A similar diminution in the measurement takes place if the thigh be flexed, but is much more marked. In order that the comparative length of the limbs may be accurately estimated it is necessary, therefore, to place them as nearly as possible in corresponding positions before the measurement is taken. The amount of shortening thus estimated is known as *true shortening*, and, provided no dislocation is present, is due to diminished length of the femoro-tibial shaft caused by (*a*) destruction of bone, *e.g.* tubercle; (*b*) overlapping or impaction, *e.g.* fracture; (*c*) arrested development, *e.g.* infantile paralysis. A corresponding condition of true lengthening may arise from long-standing inflammations affecting the bone. Examples are met with in epiphysitis and congenital syphilis. In the latter condition lengthening may be considerable, as shown by a case now under the writer's observation in which the increase amounts to $1\frac{3}{4}$ inch.

Neither lengthening nor shortening may be obvious when the patient is recumbent, and provided the disparity is not too great the legs may appear equal in length. It follows then that lengthening or shortening can be masked by posture so that the feet occupy the same plane.

In order that this may take place free movement at both hip joints is necessary.

As, however, the fact that the feet occupy the same plane is no proof that the limbs are of equal length, so the fact that one is "apparently" shorter is no proof that such is actually the case. As real shortening or lengthening can be masked by posture, so, reversely, can posture give rise to a condition of "apparent" shortening or lengthening. In this

Adduction Responsible for Apparent Shortening.

Abduction Responsible for Apparent Lengthening.

case one hip must be fixed. In some cases both hips are fixed.

Three factors must be noted in this relation between length and posture:—

(1) Maintenance of parallelism brought about by

(2) Tilting of the pelvis and consequently of the sacrum.

(3) Compensatory lateral curvature of the spine in order that the centre of gravity may fall within the base of support.

Whether the difference in length be real or apparent the pelvis is tilted as reference to the diagrams will show. In its relation to lateral curvature, therefore, apparent shortening is as active a mechanical factor as real shortening, and *vice versa.* A slight lateral curve due to tilting of the pelvis may be, and often is, the commencement of true scoliosis. The sense of security must not be fostered by the fact that the shortening is only "apparent," nor must it be forgotten that true shortening may only be demonstrable by careful measurement. The term "apparent" is a bad one. "Shortening due to posture" is much more satisfactory, for in many cases apparent shortening includes a certain amount of real shortening. The amount of shortening due to posture may be estimated by subtracting the real from the apparent, and to this extent can the shortening be remedied by correction of the vicious posture assumed. The real shortening can only be remedied by artificial lengthening of the limb. In all cases of tilted pelvis the higher spine is associated with the adducted limb, the lower spine with the abducted limb.

## Figures and Tables

**Figure f1:**
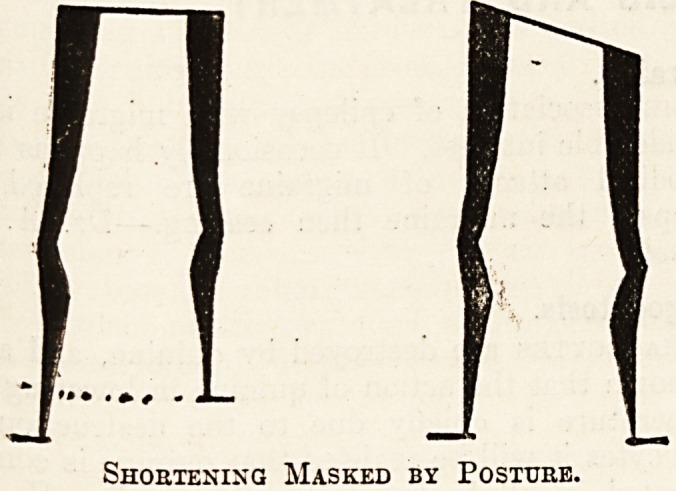


**Figure f2:**
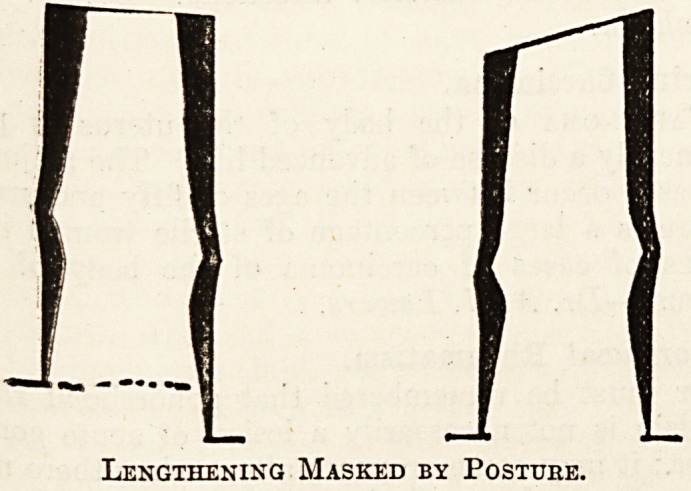


**Figure f3:**
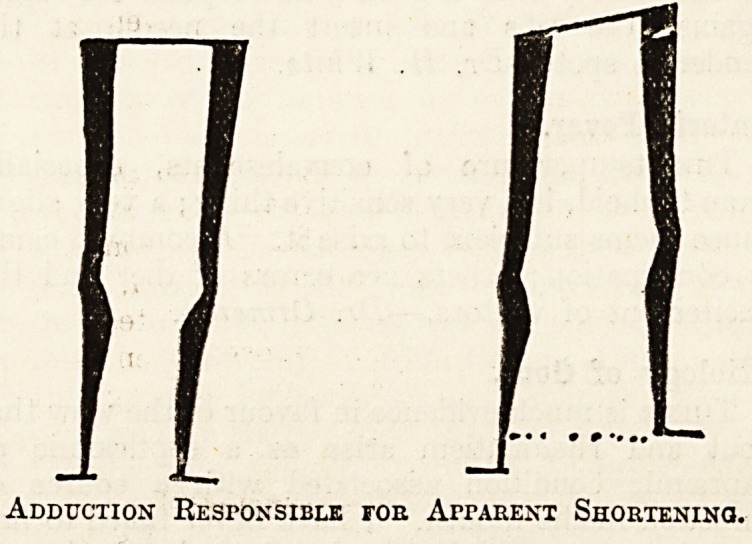


**Figure f4:**